# Toward an Automated Measure of Social Engagement for Children With Autism Spectrum Disorder—A Personalized Computational Modeling Approach

**DOI:** 10.3389/frobt.2020.00043

**Published:** 2020-04-15

**Authors:** Hifza Javed, WonHyong Lee, Chung Hyuk Park

**Affiliations:** ^1^Assistive Robotics and Telemedicine Laboratory, Department of Biomedical Engineering, School of Engineering and Applied Science, The George Washington University, Washington, DC, United States; ^2^School of Computer Science and Electrical Engineering, Handong Global University, Pohang, South Korea

**Keywords:** computational model, personalization, social engagement, autism spectrum disorder, convolutional neural network

## Abstract

Social engagement is a key indicator of an individual's socio-emotional and cognitive states. For a child with Autism Spectrum Disorder (ASD), this serves as an important factor in assessing the quality of the interactions and interventions. So far, qualitative measures of social engagement have been used extensively in research and in practice, but a reliable, objective, and quantitative measure is yet to be widely accepted and utilized. In this paper, we present our work on the development of a framework for the automated measurement of social engagement in children with ASD that can be utilized in real-world settings for the long-term clinical monitoring of a child's social behaviors as well as for the evaluation of the intervention methods being used. We present a computational modeling approach to derive the social engagement metric based on a user study with children between the ages of 4 and 12 years. The study was conducted within a child-robot interaction setting that targets sensory processing skills in children. We collected video, audio and motion-tracking data from the subjects and used them to generate personalized models of social engagement by training a multi-channel and multi-layer convolutional neural network. We then evaluated the performance of this network by comparing it with traditional classifiers and assessed its limitations, followed by discussions on the next steps toward finding a comprehensive and accurate metric for social engagement in ASD.

## Introduction

Social engagement of a child is an indicator of his/her socioemotional and cognitive states. It is the interaction of a child with the environment in a contextually appropriate manner and reflects a complex internal state that signifies the occupation of the child with a person or a task. Much of the research so far has relied on the perceptual evaluation of engagement, utilizing questionnaires and behavioral assessments administered by trained professionals, which typically attempt to identify key behavioral traits that serve as important indicators of social engagement. Automatic quantification of engagement is still limited but can allow not only for an objective interpretation of engagement and the contributing target behaviors, but also help to identify methods to improve engagement in different settings, especially when targeting a specific health condition. Therefore, it serves both as an outcome measure and as an objective measure of the quality of an activity, interaction, or intervention (Kishida and Kemp, 2006).

Social engagement has often been reported to be particularly deficient in children with Autism Spectrum Disorder (ASD). ASD is a neurodevelopmental disorder that causes significant impairment in three broad areas of functioning: communication, social interaction, and restricted and repetitive behaviors (American Psychiatric Association., 2013). This means that children interact with their peers infrequently, thus preventing the formation of lasting and meaningful social relationships and resulting in social withdrawal. These children often feel isolated from or rejected by peers and are more likely to develop behavioral problems (Ollendick et al., 1992) as well as anxiety and depression (Tantam, 2000; Bellini, 2006).

Behavioral and physiological cues can provide insight into the engagement state of a child, with gestures, subtle body language changes, facial expressions, vocal behaviors, and various physiological signals, all carrying significant indications of a child's level of interest and engagement in an interaction. Eye gaze focus, smiling, vocalizations, joint-attention, imitation, self-initiated interactions, and triadic interactions are among the important behavioral cues that can be utilized to assess engagement (Tiegerman and Primavera, 1982, 1984; Wimpory et al., 2000; Nadel, 2002; Ingersoll, 2008; Stanton et al., 2008; Katagiri et al., 2010; Sanefuji and Ohgami, 2011; Tapus et al., 2012; Slaughter and Ong, 2014; Dubey et al., 2015; Contaldo et al., 2016). Heart rate, electrodermal activity, electrocardiography, electromyography, blood pressure etc. are among the key physiological indicators of engagement state (Kushki et al., 2012; Lahiri et al., 2012; Hernandez et al., 2014). A combination of these multi-modal behavioral and physiological features can present a comprehensive feature set for effective engagement evaluation.

A major hurdle in the path toward automated measurement of social engagement is of the identification and classification of these key behaviors. While it may be a simple task for trained professionals to identify these high-level behaviors and infer a fairly accurate engagement state from real-time observations of a child's interactions, it remains a considerable challenge for the state-of-the-art algorithms and machines. Instead, the current technologies are better equipped to extract lower-level behaviors that can be used as a rough estimation of the target behaviors.

This paper presents our first step toward an automated quantifiable measure of social engagement derived from behavioral data collected from two groups of children, one typically developing (TD) and one with ASD. Research from our team thus far has focused on child-robot interaction scenarios that target several ASD symptoms, including sensory processing (Javed et al., 2019), imitation (Bevill et al., 2017), emotion recognition and emotion regulation skills (Javed et al., 2018). In these studies, we collected multi-modal interaction data, including video and audio recordings, as well as motion tracking data. The overall goal of our work is to develop a framework for personalized child-robot interactions for ASD. To this end, our framework aims to (1) sense important features of a child's interaction with a robot, (2) interpret and derive meaningful deductions about a child's engagement in the interaction, (3) identify target behaviors that may be lacking in the detected interaction pattern, (4) reassess the current robot behavior strategy and modulate it to elicit a higher level of engagement from the child. This paper focuses on step 2 of the above approach by processing the multimodal behavioral data collected from this study through a deep learning-based multi-label classification model in order to contribute toward deriving an automated measure of social engagement.

This paper is organized as follows. Section Related Work discusses the previous studies that have designed methods to formulate an automated measure of social engagement. Section Interaction Scenario Design describes the child-robot interaction scenario we used in this study. Sections Multimodal Data Collection and Extracting Ground Truth present the modalities of the data we collected during our experiments and the methods we employed to label these data. Sections Feature Extraction and Network Architecture discuss our feature extraction methods and design of our convolutional neural network for multi-label classification. Sections User Study, Results, and Comparison with Other Machine Learning Classifiersdescribe the user study, its results and a comparison of the proposed network with other classical algorithms. Section Discussion presents a discussion on these findings while Section Conclusion concludes this paper with comments on the future work.

## Related Work

Several studies in the past have contributed to this area of research with each method typically varying in terms of the feature set, number of engagement classes and computational model that were used, as well as the demographics of the participants from whom the data were collected. Rajagopalan et al. (2015) showed the feasibility of utilizing low-level behavioral features in the absence of accurate high-level features, and used a two-stage approach to first find hidden structures in the data (using Hidden Conditional Random Fields) and then learn them through a Support Vector Machine (SVM). Only head pose orientation estimates were used to assess engagement and the approach was evaluated by conducting experiments on labeled child interaction data from the Multimodal Dyadic Behavior Dataset (Rehg et al., 2013), obtaining an accuracy of around 70%.

Gupta et al. (2016) designed an engagement prediction system that utilized only the prosodic features of a child's speech as observed during a structured interaction between a child and a psychologist involving several tasks from the Rapid ABC database. Three engagement classes and two levels of prosodic features (local for short-term and global for task-wide patterns) were defined. The system achieved an unweighted average recall of 55.8%, where the best classification results were obtained by using an SVM that utilized both categories of the prosodic features. Another study by Lala et al. (2017) used several verbal and non-verbal behavioral features, including nodding, eye gaze, laughing and verbal backchannels. The authors collected their own dataset comprising audio and video recordings based on conversational scenarios between a human user and a humanoid robot, while human annotators provided labels to establish ground truth. A Bayesian binary classifier was used to classify the user as engaged or not engaged and obtained an AUC (area under the precision-recall curve) score of 0.62.

A study from Castellano et al. (2009) used both behavioral features from the user (gaze focus and smiling) and contextual information from the activity in order to train a Bayesian classifier to detect engagement in users for a child-robot interaction scenario. The labels generated from human coding were based only on the two user behaviors. The authors reported only a slight improvement in the classifier recognition rate when using both behavioral and contextual features (94.79%) vs. when only behavioral features were utilized (93.75%), highlighting the key importance of the behavioral information.

Kim et al. (2016) investigated the use of vocal/acoustic features in determining child engagement in group interaction scenarios. The annotation scheme involves the giving and receiving of attention from other group members. They used a combination of ordinal regression and ranking with SVM to detect engagement in children and found this technique to outperform classification, simple regression and rule-based approaches. Such a system may be acceptable to use with typically-developing children, but since children with ASD may often be non-verbal and/or shy or unwilling to communicate using speech/vocalizations, the exclusive use of acoustic features may not be suited to research involving the ASD population.

Another study from Parekh et al. (2018) developed a video system for measuring engagement in patients with dementia, which uses deep-learning based computer vision algorithms to evaluate their engagement in an activity to provide behavior analytics based on facial expression and gaze analysis. Ground truth was extracted through scoring performed by human annotators by classifying engagement states in terms of attention and attitude. The video system presented in this study was exclusively tested with elderly patients with dementia who were required to participate in a digital interaction while seated directly in front of the camera. Additionally, since only facial expressions and gaze features were utilized, the proximity of the participants to the camera was important, hence, limiting their physical movements.

Oertel and Salvi (2013) studied the relation between group involvement and individual engagement using several features of eye gaze patterns defined as presence, entropy, symmetry and maxgaze. They utilized the Stockholm Werewolf Corpus, which is a video dataset of participants engaging in a game that involved the use of speech and eye gaze. Once again, since only eye gaze patterns were used as features to train a classifier, participants were required to remain seated in front of the cameras.

A study that specifically tested their system on the ASD population was from Anzalone et al. (2015) that used a combination of static (focus of attention, head stability and body posture stability) and dynamic (joint attention, synchrony, and imitation) metrics within two distinct use cases including one where the robot attempts to learn the colors in its environment with the help of a human, and another that elicits joint attention from participating children with ASD. The features were extracted using histogram heatmaps and clustered using the K-means algorithm.

In Rudovic et al. (2018) also targeted the automated measurement of engagement for ASD children with multimodal data collection including features from video (facial expressions, head movements, body movements, poses, and gestures), audio, and physiological (heart rate, electrodermal activity, and heart rate) data. The child-robot interaction setting involved an emotion recognition activity with a humanoid robot that required children to be seated in front of the robot (Rudovic et al., 2017). Participating children belonged to one of two cultures (Eatsern European and Asian) and the cultural differences were also taken into account during engagement estimation. The authors generated ground truth through expert human labelers who marked changes in engagement on a 0–5 Likert scale that is based on the different levels of prompting required from the therapist during the interaction with the robot. In fact, in this work, child engagement is considered to be a function of task-driven behavioral engagement and affective engagement.

Despite the overlap, this approach is significantly different from the one proposed in this paper in several ways. Firstly, we define engagement as a function of several key behavioral indicators that provide an insight into an individual's internal engagement state (Javed et al., 2019), which generates a novel measure to estimate social engagement state i.e., the engagement index. Additionally, our methods do not restrict the movement of the subjects by requiring them to be seated in front of a camera or a robot, and the interaction design allows for free, naturalistic movement in order to closely resemble real-world social settings as opposed to other restrictive experimental approaches. Importantly, this approach toward engagement estimation can be easily generalized to any child, with or without ASD, and to a variety of different, interactive experimental settings that may or may not involve a robot.

The work described in this paper presents a social engagement prediction system for children. It utilizes a combination of features extracted from facial expressions and upper body motion tracking data to train a deep convolutional neural network that can then classify the engagement state of a child. We intentionally designed the experiments to not be strictly structured in order to encourage naturalistic and unguided child-robot interactions during data collection that impose no restrictions on the movement of a child. The nature of the features used in our approach allow for independence of interaction context and can easily be extended to a variety of scenarios within laboratory or home settings. In addition, a unique engagement model is obtained for every individual participant to ensure personalized interaction with the robot, giving it potential to be used as an intervention tool for ASD.

## Interaction Scenario Design

For this work, we used socially assistive robots to design a child-robot interaction that targeted the sensory processing difficulties in ASD, as detailed in our previous work (Javed et al., 2019). In this pedagogical setting, two different mobile robots were used to model socially acceptable responses to potentially overwhelming sensory stimulation that a child is likely to encounter in everyday experiences. The humanoid robot, Robotis Mini (from Robotis) and the iPod-based robot, Romo (from Romotive) both had their unique set of capabilities. While Mini used gestures and speech to communicate, Romo relied mostly on its large set of emotional expressions and some movements.

**Figure 1:**
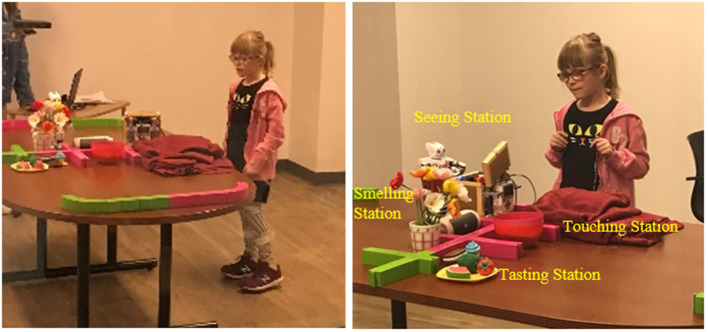
Station setup for the sensory maze game (the child's photo rights reserved).

**Figure 2:**
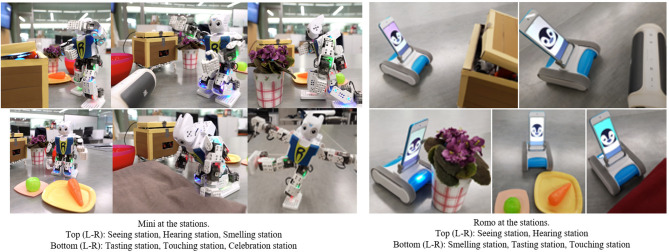
The two robots at each sensory station, adapted from Javed et al. (2019).

A maze-like setup consisting of a station for each of the visual, auditory, olfactory, gustatory, tactile and vestibular senses was used, as shown in Figure 1. Though one of the goals of the interaction was to leverage the relationship between a robot and a child with ASD, as established by a plethora of previous research (Dautenhahn and Werry, 2004; Scassellati, 2007; Diehl et al., 2012; Cabibihan et al., 2013), the focus of this work (Javed et al., 2019) was to assess the potential of this setup as a tool to socially engage children with ASD and to use the collected data to contribute toward deriving an automated measure of social engagement. Each sensory station simulated an everyday experience, such as encountering bright lights at the *Seeing station*, loud music at the *Hearing station*, scented flowers at the *Smelling station*, different food items at the *Tasting station*, materials with different textures at the *Touching station* and summersaulting to celebrate at the vestibular station (Figure 2). These scenarios were chosen to incorporate everyday stimulation that all children experience in uncontrolled environments like malls, playgrounds, cinemas etc. and in the activities of daily living such as eating meals and dressing. This interaction was designed to be highly interactive and engaging, and required the child to participate actively by answering questions from the robots, following their instructions, and “helping” them complete the maze. Details of this study, including the nature of interaction between the children and the robots, can be found in Javed et al. (2019).

## Multimodal Data Collection

A high-quality measure for social engagement estimation must take into account all behavioral and physiological cues that can serve as quantifiers of social motivation and social interaction. As discussed in Section Introduction, a number of behavioral traits and physiological signals can be used effectively to this end. However, when designing an interaction for autistic children, their unique needs and sensitivities must be taken into account. For this study, this meant that only non-contact sensors could be used in order to limit tactile disturbances to the children and enable free movement to allow for naturalistic interaction. The combination of sensors also needed to provide a wholistic and accurate representation of a child's engagement changes over the length of the interaction.

**Figure 3:**
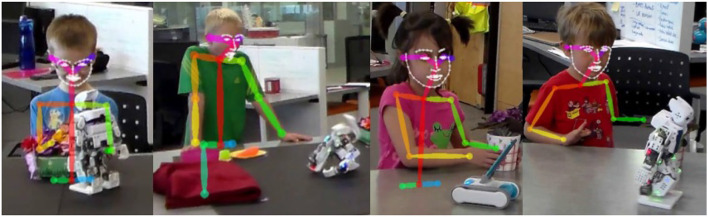
Upper body and facial keypoints generated by OpenPose.

We collected video recordings of the child-robot interactions with a camcorder placed in one corner of the room, which was repositioned by an instructor as the child moved during the interaction. From these recordings, we were able to extract audio data as well as 2-D motion tracking data with the OpenPose library (Cao et al., 2017). While OpenPose provides full body motion tracking (Figure 3), we were only able to utilize upper body data since the chosen experimental setting meant that children were often standing in front of the table that hosted the maze setup, preventing a full-body view from being captured. In addition, OpenPose also allowed for the extraction of facial expression datapoints from the same video data.

## Extracting Ground Truth

Unlike some of the previous studies described in Section Related Work, we did not use any existing video datasets to test our methods. Since our goal was to derive an engagement measure specific to the interactions that we designed for children with ASD, we opted to test our methods on the relatively limited data available from our user study. To extract ground truth for a child's engagement in the interaction with the robots, we defined six target behaviors that have been found to be key behavioral indicators of social engagement (Tiegerman and Primavera, 1982, 1984; Wimpory et al., 2000; Nadel, 2002; Ingersoll, 2008; Stanton et al., 2008; Katagiri et al., 2010; Sanefuji and Ohgami, 2011; Tapus et al., 2012; Slaughter and Ong, 2014; Dubey et al., 2015; Contaldo et al., 2016). These included eye gaze focus, vocalizations, smiling, self-initiated interactions, triadic interactions and imitation.

Three raters then coded these videos using the Behavioral Observation Research Interactive Software (BORIS) (Friard and Gamba, 2016) to annotate the start and stop times of each target behavior as it was identified in the video recordings. An inter-coder correlation (ICC) score of 0.8752 ± 0.145 was achieved for the 18 participants, which was used to evaluate the quality of the annotations. Details of the evaluation criteria are reported in Javed et al. (2019).

An eye gaze event was tagged each time the child's gaze moved to the robots or the setup and stopped when the gaze focus was lost. Vocalizations comprised of any verbal expression from the child, including but not limited to a shriek of excitement while interacting with the robots or the utterance of words to communicate sentiments or queries regarding the robots. Smiling recorded all events where a child was observed to visibly express joy in the form of a smile or laugh. Self-initiated interactions involved all interactions with the robots or setup that are initiated by the child. Triadic interactions comprised of an interaction where a child voluntarily involved a third entity in the interaction with the robot, such as sharing their excitement with the parent. Lastly, imitations included all events of voluntary imitation the robot's actions by the child. An in-depth report on the inclusion criteria of the target behaviors, their significance and annotations in video data can be found in Javed et al. (2019).

**Figure 4:**
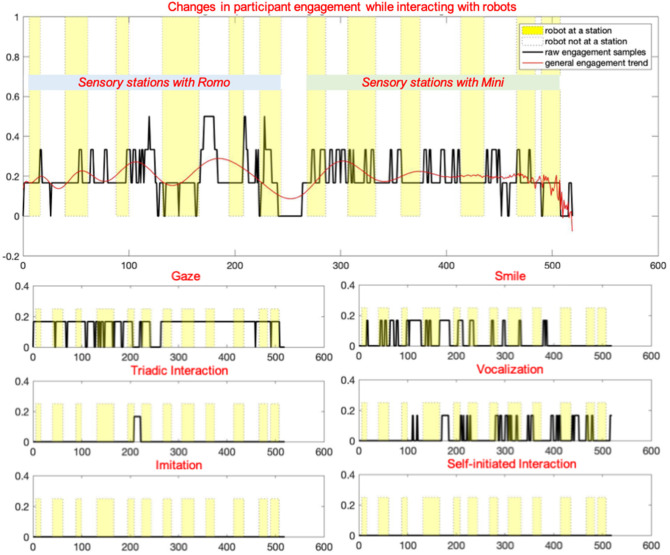
Plots depicting changes in the overall engagement level of a child during an interaction, along with subplots of the target behaviors contributing to this engagement, adapted from Javed et al. (2019).

Based on these annotations, multiple analytics were derived to quantify the social engagement with respect to each robot and target behavior, and across stations to obtain a fine-grained analysis of the child's interaction preferences (Javed et al., 2019). However, for the current work, we have only used the raw time series data of every child's changing engagement state as determined by the chosen target behaviors. These overall engagement changes are shown in Figure 4, along with the subplots of each contributing key behavior.

Therefore, each instance of time was mapped to an engagement state. Every behavior contributed a factor of 1/6 to the engagement value, thus resulting in a metric with seven distinct values that ranged from 0 (no target behavior observed) to 1 (all target behaviors observed).

## Feature Extraction

An ideal automated engagement measure in this case would incorporate all of the above behaviors, but also necessitates the automated classification of these behaviors. This is no trivial task, and involves contributions from multiple disciplines including computer vision, speech analysis and machine learning. As a part of a more practical approach that is fitting of a first step toward the derivation of an automated measure of social engagement in ASD, we decided to extract low-level behavioral components from our video data as indicators of engagement in the interactions with the robots. For this purpose, we utilized the 2D body tracking and facial expression data generated by OpenPose (Cao et al., 2017).

Using the body tracking data, we derived three new features based on Laban Movement Analysis (LMA), a method for describing and interpreting all types of human movement (Groff, 1995) used frequently in a variety of fields including dance, acting, music, and physical therapy etc. LMA categorizes all body movements into the categories of body effort, space and shape. Out of the four categories, effort represents the dynamics of human movement and provides an insight into the subtle characteristics of movements with respect to inner intention. This makes it an important feature to use in studies involving the estimation of affect, intention, and engagement states. Effort itself is classified into space, weight and time, which are the three features that we incorporated in our current work. Space represents the area taken up over the course of a movement, weight indicates the power or impact of movement, and time conveys the speed of an action, including a sense of urgency or a lack thereof in a movement. The equations (Masuda et al., 2009; Wakayama et al., 2010) for each of these features are as shown in Table 1.

**Table 1:** Equations for the derived Laban features adopted from Masuda et al. (2009) and Wakayama et al. (2010).

**Feature**	**Equation**	
Space		$space=\left(0.5|\overset{⇀}{a}||\overset{⇀}{d}|\sin\left(\theta_{1}\right)\right)+\left(0.5|\overset{⇀}{c}||\overset{⇀}{b}|\sin\left(\theta_{2}\right)\right)$
	where	$\overset{⇀}{a}$ = *Position vector from left shoulder to left hand*
		$\overset{⇀}{b}$ = *Position vector from right shoulder to left shoulder*
		$\overset{⇀}{c}$ *= Position vector from right hand to right shoulder*
		$\overset{⇀}{d}$ *= Position vector from left hand to right hand*
		*θ_1_ = Angle between* $\overset{⇀}{a}$& $\overset{⇀}{d}$
		*θ_2_ = Angle between* $\overset{⇀}{c}$& $\overset{⇀}{b}$
Weight		*$Weight=\underset{i}{\sum }\tau_{i}\left(t\right)$*
	where	*τ_*i*_ = L${}^{2}\omega_{i}$*^2^sin(θ) *mass
		*$\omega_{i}=\frac{d\theta }{dt}$*
		*L = Distance between joints*
		*i = Joint number*
		*${\dot{\omega }}_{i}$ = Angular velocity for joint i*
Time		$Time_{i}=\underset{i}{\sum }{\dot{\omega }}_{i}\left(t\right)$
	where	*i = Joint number*
		*${\dot{\omega }}_{i}$= Angular velocity for joint i*

**Figure 5:**
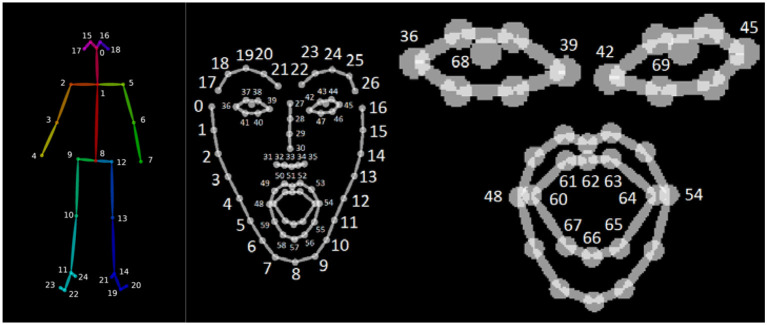
Illustrations of the skeletal and facial keypoints extracted by OpenPose (CMU Perceptual-Computing-Lab, 2019) (permission acquired from the author for using these images with citation).

OpenPose generates 50 keypoints for skeletal tracking as described in Cao et al. (2017). In addition to the skeletal data, we also recorded facial keypoints to incorporate the changes in a child's facial expressions in our feature set. Figure 5 [taken from CMU Perceptual-Computing-Lab (2019)] depicts these datapoints. While a total of 69 facial keypoints is available, we only used the lip and eye keypoints shown on the right. Including the x and y coordinates for each of the 34 facial keypoints and the three Laban features derived from the upper body skeletal keypoints created a total of 71 features in the dataset. A moving window of 1 s, i.e., 30 frames, was used to compute the Laban features in order to incorporate the sequential nature of the movement data. A 1 second interval was chosen to capture meaningful, yet rapidly changing movement patterns in response to the actions of the robot during the child-robot interaction. The number of available datapoints per participant depended on the length of interaction of each participant and ranged between 9,300 and 30,508 datapoints. Further details are listed in **Table 3**.

## Network Architecture

**Figure 6:**
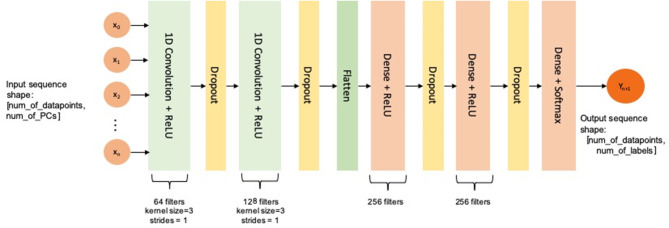
Architecture of the CNN used for multi-label classification.

We used a multi-channel and multi-layer convolutional neural network (CNN) for this temporal multi-label classification problem. The network was composed of two Conv1D layers to identify temporal data patterns (with 5 channels with 64 and 128 filters, respectively, and a kernel size of 3 with 20% dropout) and three dense layers for classification [kernel sizes 256, 256, and 7 (number of output labels: value ranges of engagement level)]. This is illustrated in more detail in Figure 6. A 10-fold cross-validation (train/test split of 0.8/0.2) was used for every subject's individual dataset and optimization was performed using the Adam optimizer.

The two Conv1D layers are meant to extract high-level features from the temporal data since the dataset being used has a high input dimension and a relatively small number of datapoints. Since the data have a non-linear structure, the first two dense layers are used to spread the feature dimension, whereas the last one generates the output dimension. The dropout layers are used to avoid overfitting.

## User Study

We conducted a user study with a total of 18 children, 13 TD and 5 with ASD between the ages of 4 and 12 years who participated in a one-time interaction with our robots within the setting of a sensory maze game. The average age of the TD group was 7.07 ± 2.56 years and that of the ASD group was 8.2 ± 1.10 years. The TD group consisted of 5 females and 8 males, whereas the ASD group was composed of all male participants. These details are presented in Table 2.

**Table 2:** Demographic details of the subjects.

**ID**	**Age**	**Gender**	**Group**
1	10	M	TD
2	4	F	TD
3	5	F	TD
4	11	F	TD
5	9	M	TD
6	10	F	TD
7	9	M	TD
8	5	M	TD
9	5	F	TD
10	5	M	TD
11	5	M	TD
12	5	M	TD
13	9	M	TD
14	7	M	ASD
15	8	M	ASD
16	10	M	ASD
17	8	M	ASD
18	8	M	ASD

The participants were allowed to participate for the entire course of the interaction as designed with the two robots, one after another. The data presented in this study is for one-time interactions between each subject and the robots. The length of the interaction for each participant is listed in Table 2. The average TD interaction length was 464.92 s whereas that of the ASD group was 620 s. Individual engagement prediction models were generated for each participant and their performances were evaluated.

## Results

Table 3 presents the detailed results produced by training, validation and testing our network for every subject in the study. The length of interaction is important and provides an insight into the number of video frames, and hence, the datapoints that would be available to the network. The datapoint count is also affected by the processing performed by OpenPose, which can drop some frames where processing could not be completed. This is particularly evident in the case of participant 6 and 12, where the number of available datapoints are far fewer than expected.

**Table 3:** Performance metrics for the individual classifiers (TD Group: ID1–ID13, ASD Group: ID14–ID18).

**ID**	**Interaction length (s)**	**No. of datapoints (frames)**	**Train**		**Validation**		**Test**
			**Accuracy**	**Loss**	**Accuracy**	**Loss**	**Accuracy**
1	315	9,444	0.8101	0.5028	0.7790	0.6681	0.7946
2	519	15,357	0.6499	0.7278	0.6398	0.7797	0.6393
3	540	16,412	0.6703	0.8723	0.6407	1.0095	0.6526
4	658	10,933	0.8302	0.4189	0.8131	0.4923	0.8240
5	797	22,996	0.9255	0.1903	0.9198	0.2484	0.9159
6	696	9,300	0.9200	0.2850	0.8925	0.3856	0.9124
7	316	9,388	0.7821	0.5423	0.7417	0.7946	0.7338
8	457	13,725	0.7561	0.6065	0.7418	0.6796	0.7483
9	574	10,463	0.6671	0.8486	0.6535	0.9333	0.6364
10	780	16,627	0.9104	0.2253	0.8831	0.3907	0.8698
11	726	12,726	0.8390	0.3843	0.8303	0.4039	0.8283
12	685	9,723	0.8118	0.5162	0.7715	0.6980	0.7720
13	540	12,879	0.8084	0.4296	0.7812	0.5858	0.7702
14	517	15,502	0.8163	0.4417	0.7952	0.5621	0.7907
15	578	14,624	0.9204	0.2276	0.8923	0.3390	0.9108
16	679	15,950	0.6810	0.7582	0.6501	0.9095	0.6398
17	610	16,401	0.8306	0.3946	0.8232	0.4923	0.8366
18	1058	30,508	0.7822	0.5467	0.7759	0.6323	0.7812

Before presenting the results, it must be highlighted that the metrics shown in this work are all weighted metrics, so as to address the impact of the imbalance in engagement level samples within the dataset. The network has an average accuracy of 0.7985 for the TD group and 0.8061 for the ASD group in the training stage. For the test data, the performance remains steady with an average accuracy of 0.7767 for the ASD group and 0.7918 for the TD group. These details are shown in Table 4.

**Table 4:** Average metrics to compare classifier performance.

**ID**	**Average interaction length (s)**	**Train**		**Validation**		**Test**
		**Accuracy**	**Loss**	**Accuracy**	**Loss**	**Accuracy**
TD	584.8	0.7985	0.5038	0.7760	0.6207	0.7767
ASD	688.4	0.8061	0.4738	0.7873	0.5870	0.7918

**Figure 7:**
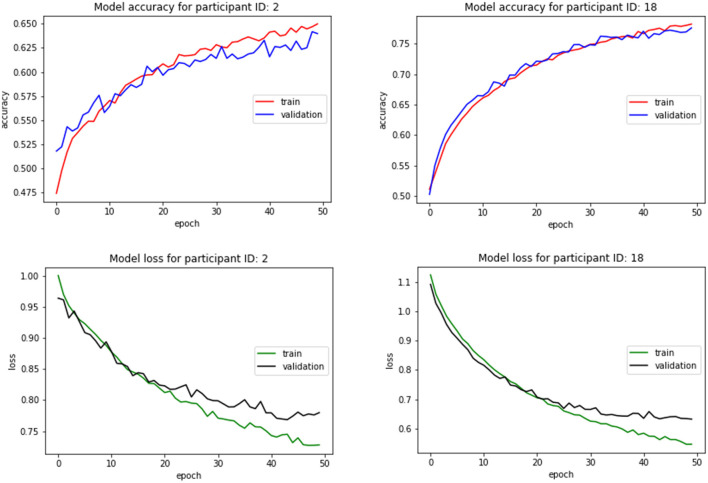
Classifier accuracy and loss with respect to the number of epochs for two different participants.

**Figure 8:**
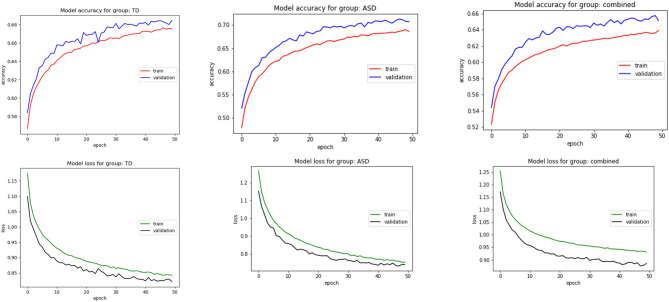
Plots showing the ground truth labels in red and the classifier predictions in blue.

Figure 7 depicts the accuracy and loss plots for training and validation data for a participant from each group illustrating the changes in accuracy with respect to the number of epochs. Figure 8 shows the timeseries plots of the changing engagement states for the participants. The red line shows the true engagement as determined by the annotations (Javed et al., 2019). Predictions made by the network are marked in blue. Since the dataset was randomly partitioned into test and training data, the predictions on the test set appear as a scatter plot.

In addition to the individual models described above, we also trained a group model for each of the two groups by using all the datapoints collected from the participants from each group. The ASD classifier was able to achieve a training accuracy of 0.6389 and a test accuracy of 0.6524, while the TD classifier achieved a slightly higher training accuracy of 0.6733 and a test accuracy of 0.6803. The slightly superior performance of the classifiers on the test data as opposed to the training data can be attributed to the use of regularization techniques used when constructing the classifier structure, in this case, the Dropout layers, which are only applied during the training phase.

**Figure 9:**
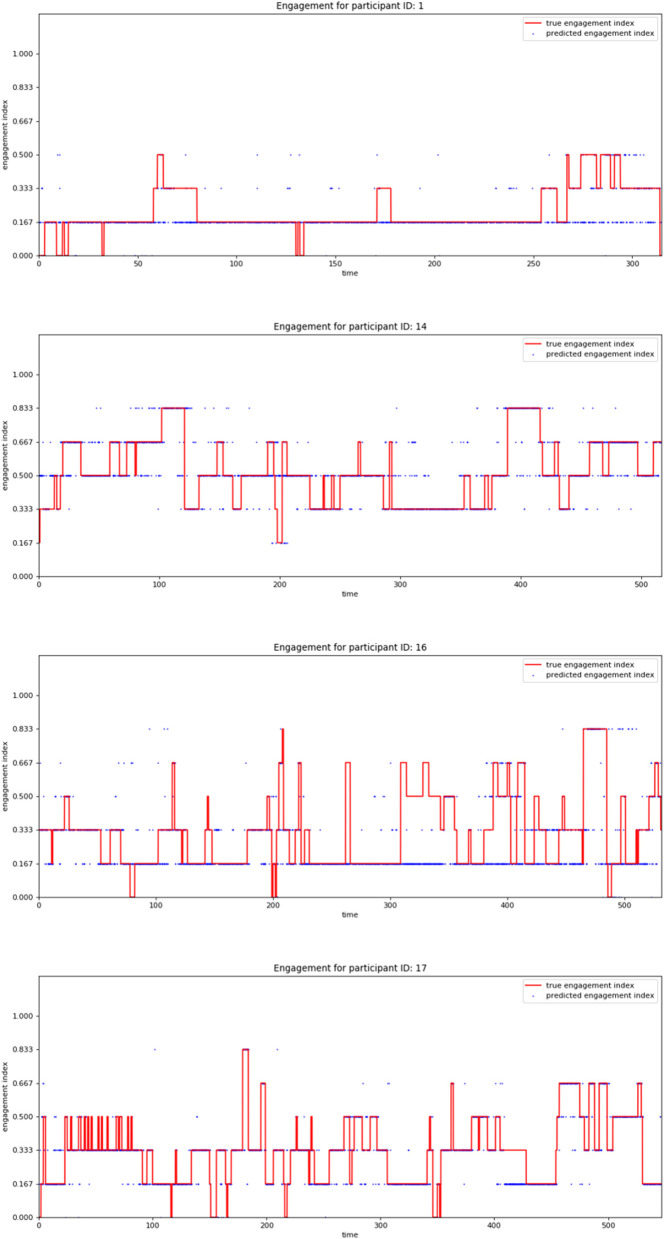
Classifier accuracy and loss for training and test datasets for three grouped conditions.

We also trained a combined classifier on the data collected from all the participants. This model underperformed slightly compared to the group-specific classifiers, indicating that a group-specific classifier may be better suited for generalization to all participants within the group rather than a single classifier for all participants (Table 5). Accuracy and loss plots for the training and validating processes for all three grouped conditions are shown in Figure 9.

**Table 5:** Performance metrics for group classifiers.

**Classifier**	**Train**		**Validation**		**Test**
	**Accuracy**	**Loss**	**Accuracy**	**Loss**	**Accuracy**
TD	0.6733	0.8472	0.6800	0.8263	0.6803
ASD	0.6389	0.9320	0.6512	0.8858	0.6524
Combined	0.6733	0.8472	0.6800	0.8263	0.6803

## Comparison With Other Machine Learning Classifiers

A number of standard Machine Learning (ML) classifiers were also trained for all the scenarios described above as a way to situate the performance of the CNN, which included Support Vector Classification (SVC), Random Forest (RF), Decision Trees (DT), and K-Nearest Neighbors (KNN). The reported metrics were also averaged across all participants to compare the overall performance of the classifiers. As before, each classifier was trained and tested on entire group datasets to compare performance as a generalized group classifier. These results are shown in Table 6.

**Table 6:** Performance metrics for all classifiers under individual and group conditions.

	**Classifier**									
	**CNN**		**SVC**		**RF**		**DT**		**KNN**	
**ID**	**Accuracy**	**F1**	**Accuracy**	**F1**	**Accuracy**	**F1**	**Accuracy**	**F1**	**Accuracy**	**F1**
1	0.79	0.77	0.77	0.72	0.80	0.78	0.77	0.75	0.81	0.79
2	0.64	0.62	0.58	0.55	0.75	0.75	0.65	0.64	0.72	0.71
3	0.65	0.59	0.66	0.55	0.67	0.61	0.65	0.58	0.67	0.61
4	0.82	0.79	0.82	0.76	0.83	0.81	0.82	0.79	0.83	0.81
5	0.92	0.91	0.89	0.87	0.93	0.92	0.90	0.89	0.93	0.93
6	0.91	0.89	0.92	0.90	0.90	0.89	0.91	0.89	0.92	0.90
7	0.73	0.73	0.61	0.59	0.80	0.80	0.72	0.71	0.80	0.80
8	0.75	0.74	0.51	0.47	0.82	0.82	0.66	0.66	0.82	0.81
9	0.64	0.57	0.63	0.56	0.65	0.60	0.63	0.57	0.67	0.61
10	0.87	0.87	0.79	0.77	0.88	0.87	0.82	0.82	0.85	0.85
11	0.77	0.76	0.69	0.65	0.78	0.77	0.72	0.71	0.76	0.74
12	0.83	0.78	0.81	0.74	0.84	0.81	0.82	0.79	0.84	0.80
13	0.77	0.77	0.73	0.69	0.79	0.80	0.77	0.77	0.79	0.80
14	0.79	0.79	0.70	0.69	0.82	0.81	0.73	0.73	0.81	0.81
15	0.91	0.90	0.87	0.83	0.92	0.90	0.90	0.88	0.92	0.91
16	0.64	0.62	0.61	0.57	0.67	0.65	0.62	0.60	0.68	0.66
17	0.84	0.84	0.70	0.69	0.88	0.88	0.76	0.75	0.84	0.84
18	0.78	0.78	0.63	0.60	0.79	0.78	0.61	0.58	0.78	0.78
Average	0.78	0.76	0.72	0.68	0.81	0.79	0.75	0.73	0.80	0.79
TD	0.68	0.65	0.63	0.58	0.74	0.74	0.64	0.61	0.74	0.73
ASD	0.72	0.71	0.60	0.58	0.77	0.76	0.61	0.60	0.76	0.76
Combined	0.65	0.62	0.59	0.54	0.74	0.71	0.60	0.56	0.71	0.71

After averaging over the metrics for all participants, RF is seen to have the best performance followed by KNN and CNN, respectively. A similar trend is seen for grouped classifiers, where RF once again outperforms all other classifiers in terms of both the accuracy and the F1 score, followed again by KNN and CNN, respectively. All classifier performances drop slightly when data from the two groups are combined, suggesting that a single classifier may not be as useful for generalization as a group-specific classifier.

## Discussion

In this work, we propose the use of a Deep Learning Convolutional Neural Network to model and predict child social engagement as a part of our larger goal to personalize child-robot interactions. We utilized key social behaviors as indicators of engagement in an interaction, which formed the criterion for the human-generated labels that serves as the ground truth for this engagement classification approach.

We found that the proposed CNN was able to achieve a performance that was comparable to the highest performing classical ML approaches in this work. The RF and KNN classifiers only slightly outperform the CNN in the case of both individual classifiers and grouped classifiers. The individual classifiers serve as personalized engagement prediction networks for the unique behavioral expressions of each individual participant, whereas the grouped classifiers were used to evaluate the potential for a single classifier to generalize the learnt patterns to all the participants within a group.

On the individual level, the CNN was able to attain a best case accuracy of 0.92 (participant 5) and a worst case accuracy of 0.64 (participant 2). On the other hand, the RF classifier reached a highest accuracy of 0.93 (participant 5) and lowest accuracy of 0.65 (participant 9). For the averaged metrics as well as the grouped metrics, the RF accuracy is no more than 2% higher than that of the CNN.

The individual ASD and TD classifiers were generally found to achieve a higher accuracy than the single classifier trained on data from all the participants. This points the possibility of a generalized group classifier that can be used effectively to classify social engagement for all the children in each group while providing a high level of personalization in the interaction.

The CNN is a complex structure with a large number of tunable parameters that generally requires much larger datasets to fully exploit the potential of deep networks. Given the number of input features, the number of output classes and the size of the dataset (generated by single session child-robot interactions only) used in this study, the CNN was able to achieve a performance comparable to simpler ML classifiers but not exceed them. We anticipate that as we continue to collect interaction data from additional participants for a long-term study involving multiple sessions, the proposed deep learning network will likely become a more suitable choice for social engagement classification.

It must also be pointed out that in terms of deployment to a robotic platform, a CNN may also be a more suitable option since the traditional algorithms require expensive resources when deployed to mobile platform in real-world applications, whereas deep learning algorithms can fully take advantage of the scalable computing platforms with GPUs that have low-cost modules (like the NVidia Jetson Nano) while retaining the capacity to handle much larger datasets.

The current work is limited in that it only utilizes single session data for each participant based on which the classifiers are trained. Classifier performance is likely to improve as subsequent sessions are conducted and larger datasets are collected. Another limitation of this work is that the datasets for the two groups are unbalanced, with 13 participants in the TD group and only five in the ASD group generating much larger training dataset for the TD classifier than ASD. Conducting long-term studies with a population such as ASD remains a considerable challenge for all researchers in the field and explains the lack of open multi-modal datasets to benefit the ASD research community.

Since our focus in this work was to evaluate social engagement in a naturalistic interaction setting, the video recordings of the sessions mainly focused on the participant but also included other members of the research team and/or parent in several segments of the videos as the child moved around the room to interact with the robots. OpenPose was chosen to process the movements of the participants particularly because it offers a feature to track multiple persons by assigning each a fixed ID. In practice, however, this ID assignment was found to lack reliability, which we discovered by visualizing the participant's skeletal tracking data. In addition, we also found that the number of frames in the input video and the number of frames generated as output by OpenPose were often inconsistent, contributing to the loss of data.

It would be interesting to see how the classifier performance changes over long-term interactions between the children and robots. Child engagement is likely to vary with continued exposure to the robots and inclusion of additional temporal features in the dataset may become important. We also aim to incorporate additional modalities to our dataset, including physiological signals like heart rate, electrodermal activity, body temperature and blood pressure, as well as audio features. For this complex feature set, we foresee a deep learning network to be a more suitable classifier choice capable of identifying patterns relating to different levels of social engagement in children.

## Conclusion

In this paper, we presented a multi-label convolutional neural network classifier to formulate an automated measure of social engagement for children. To provide a personalized metric that is the best representation of the unique expression of emotion, interest and intention of each individual, we trained a separate classifier for each subject and then evaluated its performance. We designed the study to ensure the participants were not restricted in their movements at all in order to closely mimic naturalistic interactions in the real world. The use of this setting increases the complexity of data collection and analysis but enables the generalization of the presented analysis techniques to other interaction scenarios and populations, which sets this work apart from other research studies in this domain.

## Data Availability Statement

The datasets generated for this study are available on request to the corresponding author.

## Ethics Statement

The studies involving human participants were reviewed and approved by Office of Human Research, The George Washington University. Written informed consent to participate in this study was provided by the participants' legal guardian/next of kin. Written informed consent was obtained from the minor(s)' legal guardian/next of kin for the publication of any potentially identifiable images or data included in this article.

## Author Contributions

CP conceived this project, designed and developed the systems, and mentored the overall project. HJ developed and conducted experiments with CP to collect the data, trained the CNN model with the data with WL, and analyzed the results. WL designed the basic CNN structure for training and mentored on the application to the data. HJ wrote the initial manuscript. WL and CP provided comments and edits to finalize this manuscript.

### Conflict of Interest

The authors declare that the research was conducted in the absence of any commercial or financial relationships that could be construed as a potential conflict of interest.
