# Corrigendum: Toward an Automated Measure of Social Engagement for Children With Autism Spectrum Disorder—A Personalized Computational Modeling Approach

**DOI:** 10.3389/frobt.2020.00067

**Published:** 2020-05-27

**Authors:** Hifza Javed, WonHyong Lee, Chung Hyuk Park

**Affiliations:** ^1^Assistive Robotics and Telemedicine Laboratory, Department of Biomedical Engineering, School of Engineering and Applied Science, The George Washington University, Washington, DC, United States; ^2^School of Computer Science and Electrical Engineering, Handong Global University, Pohang, South Korea

**Keywords:** computational model, personalization, social engagement, autism spectrum disorder, convolutional neural network

In the original article, there was a mistake in the legend for Figure 4 as published. The legend contained an incorrect wrong reference (Kushki et al., 2012) which was added by mistake. The reference has been removed from the legend.

The authors apologize for this error and state that this does not change the scientific conclusions of the article in any way. The original article has been updated.

